# A minimal resting time of 25 min is needed before measuring stabilized blood pressure in subjects addressed for vascular investigations

**DOI:** 10.1038/s41598-017-12775-9

**Published:** 2017-10-10

**Authors:** Guillaume Mahe, Emmanuelle Comets, Aziz Nouni, François Paillard, Caroline Dourmap, Alexis Le Faucheur, Vincent Jaquinandi

**Affiliations:** 1 Unité de médecine vasculaire, centre de cardio-prévention, ESH Hypertension Excellence Centre, CHU Rennes, F-35033 Rennes, France; 2 INSERM, Centre d’investigation clinique, CIC 1414, F-35033 Rennes, France; 3 Université de Rennes 1 (Univ Rennes1), Rennes, France; 4 INSERM, IAME, UMR 1137, Université Paris Diderot, Sorbonne Paris Cité, Paris, France; 5 Centre hospitalier du Centre Bretagne, Noyal-Pontivy, France; 6 Movement, Sport and Health laboratory. EA 1274. UFR APS. Université de Rennes 2 (Rennes 2 university), Rennes, F-35000 France; 7 Department of Sport sciences and physical education, Ecole normale supérieure de Rennes, Campus de Ker Lann, Bruz, F-35170 France

## Abstract

Blood pressure (BP) measurement is a central element in clinical practice. According to international recommendations 3 to 5 minutes of resting is needed before blood pressure measurement. Surprisingly, no study has modelled the time course of BP decrease and the minimum resting-time before BP measurement. A cross-sectional bicentric observational study was performed including outpatients addressed for vascular examination. Using two automatic BP monitors we recorded the blood pressure every minute during 11 consecutive minutes. The data was analyzed by non-linear mixed effect regression. Systolic (SBP) and diastolic BPs were studied and we tested the effect of covariates on its evolution through log-likelihood ratio tests. We included 199 patients (66+/−13years old). SBP was found to decrease exponentially. Simulations based on the final model show that only half the population reaches a stabilized SBP (defined as SBP + 5 mmHg) after 5 min of resting-time while it takes 25 min to ensure 90% of the population has a stabilized SBP. In conclusion, our results and simulations suggest that 5 minutes are not enough to achieve a stabilized SBP in most patients and at least 25 minutes are required. This questions whether the diagnosis of hypertension can be reliably made during routine visits in general practitioners’ offices.

## Introduction

Hypertension is a highly prevalent disease worldwide affecting currently more than 1 billion people^1^. Various studies have shown that elevated blood pressure increases cardiovascular diseases such as stroke, myocardial infarction, and peripheral artery disease^2–5^. In 2010, high blood pressure was recognized as the major risk factor for global disease burden before tobacco smoking and alcohol use^6^. Yet, lifestyle modifications (including exercise, healthy dietary habits) and treatments can reduce and control blood pressure, and thus allowing reducing the morbidity and mortality in specific group population^4,7–11^.

The American Heart Association (AHA), the European Society of Cardiology (ESC), the European Society of Hypertension (ESH) and others have proposed recommendations for blood pressure measurement^2,11,12^. These guidelines suggest a resting time varying from 3 min to at least 5 min before blood pressure measurement^2,5,12^. Surprisingly, no study has modelled the time course of blood pressure decrease and the minimum resting time before blood pressure measurement in a population that should be screened for hypertension.

The hypothesis is that the resting time before the blood pressure measurement should be longer than 5 minutes to reach the stabilization of the blood pressure in patients^13,14^. Confirming this hypothesis could have important implication regarding hypertension screening. Indeed, following current guidelines could potentially mean that a significant number of individuals are wrongly diagnosed as hypertensive. This potential overdiagnosis could lead to spending money and giving inappropriate medications thus causing adverse events such as falls.

In this observational study, we evaluated the time course of the blood pressure decrease during the resting time before the blood pressure measurement in patients addressed at our vascular offices and the parameters explaining the blood pressure decrease during the resting time.

## Results

Between September 2014 and April 2015, 199 patients were recruited. Table 1 shows the baseline characteristics of the population studied in this analysis, which included 101 subjects in the reclining position (51%) and 98 subjects in the sitting position (49%). BP was followed on the left arm in n = 106 subjects (53%) and the right arm in n = 93 subjects (47%). Patients who came for a carotid ultrasound exam or suspected peripheral artery disease, or suspected venous disease or a cardiovascular prevention visit were 30 (20%), 32 (16%), 31 (15%), and 95 (48%) respectively. Two data were missing.

**Table 1:** Distribution of the covariates, comedications and clinical conditions in the 199 patients analyzed in the study.

Demographic covariates	Mean (SD)
Age (yr)	66.6 (13.1)
Weight (kg)	74.6 (14.2)
Body mass index (kg/m^2^)	26.9 (4.2)
Men	118 (59%)
Smoker	76 (38%)
**Clinical condition and concurrent medications**	**Mean (proportion)**
Oral antidiabetics	32 (16%)
Insuline	6 (3%)
Angiotensin-converting-enzyme inhibitors	85 (43%)
Diuretics	60 (30%)
Beta-blockers	63 (32%)
Antiplatelets	91 (46%)
Anticoagulants	21 (11%)
Lipid-lowering drugs	106 (53%)
Vasodilatators	10 (5%)
Other treatments	57 (29%)
**Medical history**	
Hypertension	114 (57%)
Dyspnea	44 (22%)
Myocardial infarction	27 (14%)
Angina	17 (9%)
Stroke	18 (9%)
Dyslipidemia	102 (51%)
Diabetes	36 (18%)
Chronic Obstructive Pulmonary Disease	9 (5%)
Sleep apnea disorder	12 (6%)
Renal insufficiency	16 (8%)
Cancer	7 (4%)
Atrial fibrillation	11 (6%)

**Figure 1:**
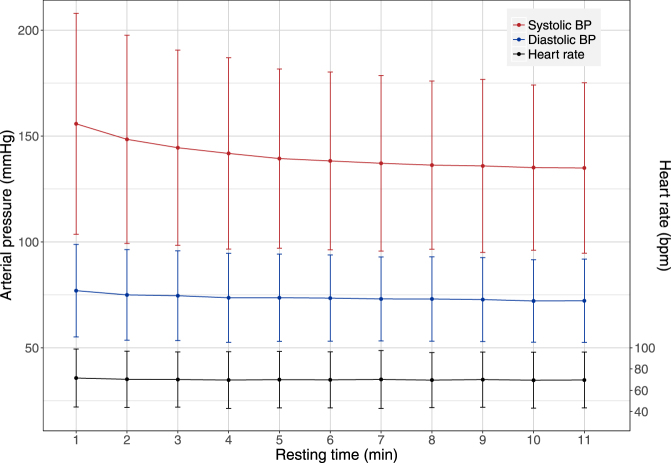
Evolution of the blood pressure and heart rate during the resting time. Evolution of measured systolic (red) and diastolic (blue) arterial blood pressure with time, expressed as mean and + /−1.96 standard deviation. Also shown, in black, is the evolution of heart rate over the measurement period (slightly displaced not to overlap the diastolic pressure); the scale for the cardiac frequency is given as the axis on the right hand side of the figure.

**Figure 2:**
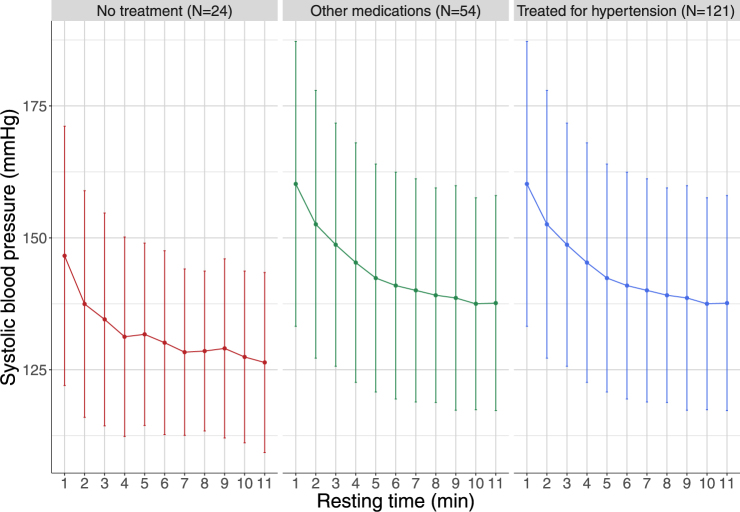
Evolution of systolic blood pressure with resting time stratified by treatment. Evolution of systolic blood pressure with resting time stratified by treatment. Left: patients receiving no chronic treatment; middle: patients receiving a treatment other than hypertensive drugs; right: patients receiving an hypertensive treatment (defined as receiving at least one drug from the following therapeutic classes: Diuretics, Betablockers or Angiotensin-converting-enzyme inhibitors).

Figure 1 shows a decreasing trend with time for SBP, which was confirmed by a repeated measure ANOVA analysis (F(1,198) = 394.4, p < 0.0001). There was also a small but steady decrease in DBP, while heart rate remained unchanged throughout the observation period. The trend for SBP was found in the absence or presence of medical treatment (Fig. 2), which shows the decrease in SBP stratified according to treatment, separating those with no treatment, those receiving drugs other than hypertensive drugs (defined as at least one diuretic, ACE inhibitor or beta-blocker drug), and those treated for hypertension. All groups exhibited the same decrease profile, but subjects who were completely free of medication tended to have lower SBP both at baseline and after resting. An exponential decrease was found to fit the data well (Appendix, Figure S3). Parameter estimates for the final model are reported in Table 2 with a systolic Prest estimated at 133 mmHg in this population. The half-life was found to be 1.7 min with large variations in the population. We did not find an impact of the position (lying or sitting) or heart rate during blood pressure measurement on the BP decrease. Baseline SBP was estimated to be 25% higher on average than systolic Prest. Age was found to be correlated both to systolic Prest and to the change from baseline. There was a large interindividual variability in the speed of decrease, as reflected by the large standard deviation on k (Table 2).

**Table 2:** Parameter estimates for final model.

Parameter	Estimate	(RSE %)	SD (%)	(RSE on IIV %)
Systolic Prest (mmHg)	133.25	(1)	13	(5)
β_Prest, Age_ (−)	0.21	(21)	—	
β_Prest, Dyspnea_ (−)	−0.097	(26)	—	
dP (−)	0.26	(4)	36	(9)
β_dP, Age_ (−)	1.15	(17)	—	
β_dP, Recent \; coffee_ (−)	0.39	(30)	—	
k (min^−1^)	0.40	(9)	89	(7)
β_k, Dyspnea_ (−)	−0.77	(24)	—	
β_k, HR_ (−)	−1.47	(28)	—	
σ (−)	0.036	(2)	—	

Prest is the asymptotic resting blood pressure representing the BP reached after a long rest, dP is the relative difference between the baseline pressure (before the subject sits or lays down), and k is a rate constant measuring the speed at which BP stabilises. RSE stands for relative estimation error, IIV denotes the interindividual variability, quantified by the standard deviation (SD) of the random effect associated to the population parameter. The β terms denote the influence of covariates on parameters, and are indexed according to the parameter and covariate names. For instance, β_Prest,Age_ denotes the effect of Age on Prest. Covariate effects were entered multiplicatively (see equations in Appendix). Continuous covariates were centered to their median value in the population (median age: 67 years old, median heart rate: 69 bpm).

**Figure 3:**
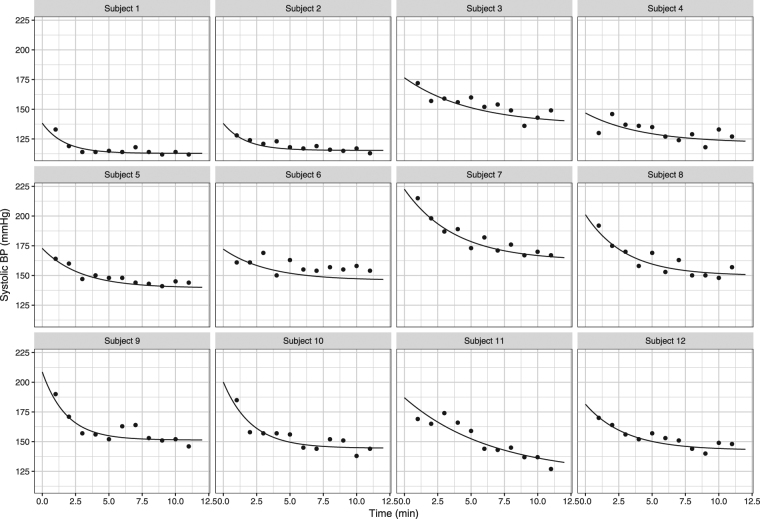
Individual model fits for 12 randomly chosen patients in the study, according to the final model. Dots represent observations and the lines are the model predictions corresponding to the empirical Bayes estimates for each subject (individual predictions).

Figure 3 shows individual model fits for 12 randomly selected patients, demonstrating a good model adequacy. Additional evaluation results can be found in the Appendix Additional results are provided in the appendix for DBP following the same procedure of analysis.

An additional analysis was undertaken to reduce the variability in k by defining a covariate reflecting the shape of the decrease. Individual non-linear regression showed that a non-linear model fit the data best in 128 subjects (64%), while 44 (22%) had a linear decrease and 24 (14%) did not exhibit significant variation of BP. This covariate was included in the model, and was found to impact both the systolic Prest and the time to stabilisation, yielding 3 groups of subjects with different values of k: fast, regular and slow stabilisers (further details in the Appendix). Taking into account individual decrease shape allowed reducing the variability in k by 40%.

### Time to reach a stable blood pressure

Simulations using the final model showed that only 50% of the population was stabilised to within 5 mmHg of systolic Prest after 5 min resting time, while up to 25 min may be needed to ensure stable BP in 90% of the population (full results in the Appendix). This resting time falls to 15.0 min if a variability of 10 mmHg is accepted.

### Proportion of the population considered as hypertensive

**Figure 4:**
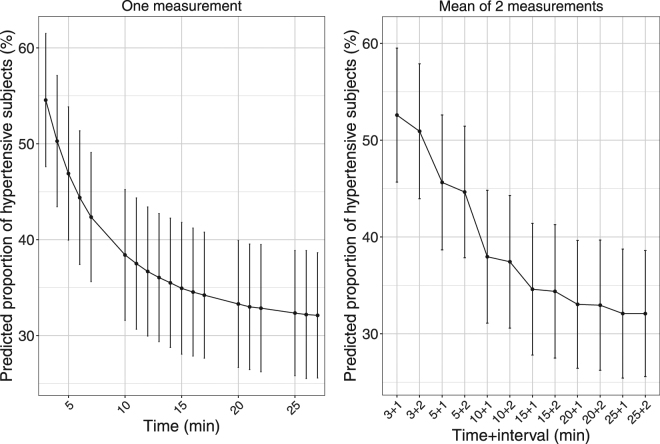
Predicted proportion of subjects diagnosed as hypertensive (systolic blood pressure above 140 mmHg) versus time of measurement. Left: single time, right: mean of 2 measurements (guidelines: 3 or 5 mn +1 or 2 mn). To obtain these figures, 1000 simulations of the original dataset were performed.

Figure 4 shows the proportion of subjects diagnosed as hypertensive depending on the number and time of the SBP measurement and its associated prediction interval. It illustrates that measurements of SBP at 3 or 5 min, single or averaged, tend to overpredict the proportion of hypertensive subjects, which stabilises only after 25 min. For example, the predicted proportion of subjects diagnosed as hypertensive drops from 50% [44–57] when averaging measurements at times 3 and 5 to 44% [38–50] 2 mn later, down to 33% [27–39] when averaging times 25 and 27 min. The width of the prediction intervals reflects the small sample size (n = 199), as we simulated replicates of the original dataset to reflect the distribution of covariates in a typical population screened for hypertension.

## Discussion

Our study showed that among outpatients addressed for a vascular examination the minimal resting time before blood pressure measurement to obtain a stable SBP_5mmHg_ in 90% of the population is 25 min. Refining the analysis by considering individual variations in the evolution of SBP, we found that there were three subgroups of patients. The regular and fast groups had the same estimated systolic Prest suggesting the “fast” group is in fact stable from the start, while slow stabilisers take much longer to reach a stable SBP than the majority of subjects.

The resting time found in this study is far longer than the resting time proposed by all previous scientific statements^5,12,15^. AHA scientific statement stated, “ideally, 5 minutes should elapse before the first reading is taken”^12^. The ESC have shortened this resting time and suggested a time between 3 to 5 minutes^15^ whereas “The Seventh Report of the Joint National Committee on Prevention, Detection, Evaluation, and Treatment of High Blood Pressure” have increased this resting time writing that “at least five minutes” were required^5^. Therefore, according to our results, the current leading guidelines about the office measurement of blood pressure might contribute to increase the prevalence of hypertension in our societies, and consequently increasing the number of patients receiving antihypertensive drugs.

The other interesting point is that we found 3 different subgroups of patients with three different patterns of pressure evolution within the resting time. The “fast” pressure group hardly needed to rest for pressure to stabilize. To obtain stable pressure for 90% of the patients in the “regular decrease pressure” group, 9 min were needed whereas up to ~53 min were needed in the “slow decrease pressure” group, although this last figure (Table S3, appendix) is extrapolated much beyond the duration of measurement in the data and would need to be evaluated with additional data. The lower systolic Prest we found for the slow group could also be due to the same reason. To our knowledge no study has shown that three different groups of patients exist for blood pressure stabilization. Unfortunately it was not possible in this study to identify specific characteristics of each group. It would be interesting to investigate this issue in a larger study, including measurements of SBP at later times as well as repeated visits to ascertain whether this finding is reproducible.

The other point raised by the study concerns the organization of the public health system to determine where and how can we diagnose hypertension. It is widely admitted that office blood pressure measurement remains the gold standard for screening^12^,^15^. Screening and diagnosis of hypertension is mainly performed by General Practitioners (GPs)^2^. However, applying our results in clinical practice at the GPs office is not realistic due to time constraints. Indeed, the mean duration of GP consultation was evaluated as 10.7 + /−6.7 minutes in six European countries^16^ and sixteen minutes in France^17^. Thus in our opinion hypertension diagnosis should be performed in a dedicated place during a specific consultation. At the very least, a suspicion of hypertension in a patient should warrant a second measurement after a longer rest period than the current recommended 5 min. Another option could be to use an out-of-office blood pressure measurement that is currently devoted to specific clinical conditions such as suspicion of white-coat hypertension or masked hypertension for example^2,18^. Even in these cases and according to our results, it seems that patients should be counseled that they have to rest at least 25 minutes and always wait the same time before blood pressure measurements. Our findings corroborate recent result showing that BP measured after a 30 mn rest is significantly lower than BP measured according to the current recommendations^19^.

Finally, when we extrapolate our results in France where ~15 million patients are treated for a hypertension we can estimate that 696 000 patients could be over diagnosed for an hypertension. This could represent an annual health public cost of 292 million euros only for the treatment cost. Furthermore giving antihypertensive drugs to patients who do not need it might also provoke drug adverse events^4^.

### Limits

Our study has several limitations. First, our population is a population of outpatients addressed to a vascular examination and not patients issued from GPs’ offices. Because they came for a regular visit, the observation period was limited to the duration of a normal consultation so we could not obtain measurement after 11 minutes, and some of our findings need to be verified using data collected in a longer study. Interestingly, our results are in accordance to a recent study from Bos and Buis^19^, who showed a significantly lower BP measured after a 30 minutes rest compared to BP measured during a routine consultation. On the other hand, the general characteristics of our population are nearly similar to those included in large randomized controlled trials on hypertension^4,9,10^. Furthermore, our outpatients came for a suspected vascular problem and should have benefited from a blood pressure measurement in order to control this cardiovascular risk factor^5,12,15^. Second, in this study, blood pressure measurements were not compared to home or ambulatory blood pressure measurements because it was not the aim of this study, and home blood pressure or ambulatory measurements are not realized in the majority of patients screened for hypertension. Third, the association of blood pressure values at specific times from baseline with cardiovascular events or target organ damage was not evaluated. As our data showed different models of blood pressure decrease upon time, it would be interesting to perform an additional study to determine which value is the best associated with cardiovascular complications. Fourth, the study included all patients addressed for a vascular appointment, and was thus not designed to test for changes in BP evolution or resting BP in populations with specific features such as comorbidities or treatment. However we did find some differences in the analysis, with older patients in particular having both higher BP and larger differences between baseline and resting BP. The model and estimated parameters from the present study could be used to design future studies investigating specific target populations^20^. Finally, repeated blood pressure measurements that induce repeated short arterial occlusion might have a role in the blood pressure decrease since transient ischemia during cuff inflation and reactive hyperemia after cuff deflation can induce dilation of upper arm artery, which is likely mediated by baroreceptor reflex and endothelium-dependent vasodilation^21,22^. However, these physiological responses are seen after occlusions that last 5 minutes^21,22^. When measuring blood pressure with the automatic device, the occlusion lasts only 10 s meaning that for 11 measurements the total duration of occlusion is less than 2 minutes. As we performed repeated occlusions, these can also mimic ischemic preconditioning^23,24^. However it has been shown that: i) there was no evidence for ischemic preconditioning during a repeated vessel occlusion that lasts 2 minutes^25^, and ii) a minimal interval of 2 minutes is needed to observe a significant effect^26^. Therefore it is not likely that such phenomenon occurs. Furthermore, Nikolic *et al*. measured the ankle SBP in 250 treated hypertensive patients at 5 and 10 mn (taking the average of two measurements 1 mn apart) and found a decrease of 4 mmHg on average between the two measurements (SD 14 mmHg), which is the same as the decrease we observed in our patients, and suggests that the additional measurements taken in our study do not impact the evolution of SBP too strongly^27^. As a final note, all international recommendations suggest to perform several measurements of blood pressure and to perform a mean of the measurements^5,12,15^.

## Conclusion

Our study suggests that the current recommended practice of measuring SBP after 5 minutes of resting may not allow for adequate stabilization of SBP, which we find could take at least 25 minutes. Public Health Policies should take into account this result to organize the best way to diagnose hypertension in our societies and avoid overdiagnosis.

## Methods

### Study design

A cross-sectional, observational, bi-center (University hospital of Rennes and Private practice in Angers) study was performed in France. The ethics review board of our institution (named “Comité d’éthique du CHU de Rennes”) approved the “Opti-PA study” (n°14.44; August 2014). All included subjects signed an informed consent. This study has been conducted according to the principles expressed in the declaration of Helsinki.

#### Study population

Outpatients (18 years of age or older), arriving on foot for a vascular examination either for a carotid ultrasound exam or suspected peripheral artery disease, or suspected venous disease or a cardiovascular prevention visit. Only patients in whom arm pressure could be measured in both arms were included. Patients with fistula or lymphedema after breast cancer were not included. Several variables were collected such as age, gender, body mass index, comorbidities and medical treatments during the medical interview or retrieved from medical record.

#### Study measurements

Demographic and clinical data were recorded at inclusion. Each patient was invited to participate in this study when coming into the office room, then the patient was invited to lie down on a bed or to seat on a chair in a controlled room temperature for the blood pressure measurements. Two cuffs adapted to the arm circumference were placed at heart level on each arm of the patient. Heart rate as well as systolic and diastolic blood pressures were automatically and simultaneously measured at each arm every minute for 11 minutes, using two Dinamaps CARESCAPE V100 (GE Healthcare®).

#### Outcome measure

The primary study outcome was the blood pressure measurements every minute for 11 minutes.

### Statistical analyses

The statistical analysis was performed first on Systolic Blood Pressure (SBP) and second on Diastolic Blood Pressure (DBP). We followed for each subject the SBP or DPB for the arm with the highest measurement at the first measurement (1 mn). The main objective was to model the decrease of blood pressure. The secondary objective was to identify the explanatory variables of this decrease, including the general characteristics of patients described in Table 1, measurement position (lying or sitting), recent coffee intake (within the previous 2 h), and mean heart rate.

#### Data analysis

Baseline pressure was the first pressure measured after 1 minute of resting. A repeated measure ANOVA analysis was first performed to determine whether a significant trend in time could be detected. The data were then analyzed through non-linear mixed effect regression using the Monolix software(see the Appendix for details on the statistical models and methods). We assumed an exponential decrease of blood pressure P(t) with time t from an initial value to an asymptotic resting pressure (Prest), according to the following equation:

$${\rm{P}}({\rm{t}})={\rm{Prest}}\,(1+{\rm{dP}}\,\exp (-{\rm{k}}\,{\rm{t}}))$$

where dP is the percentage difference in blood pressure between baseline supine pressure and systolic Prest, and k represents the rate at which the blood pressure stabilises. We assumed a log-normal distribution for the three parameters in the model and tested different residual error models to represent the measurement error. We studied the relationships between parameters and covariates. Technical details of the model building and its evaluation are provided in an Appendix, along with a similar analysis performed for DBP.

#### Prediction of the time to stabilisation

The model and parameter estimates were used to predict the time when most subjects are expected to reach a stable SBP_5mmHg_, which was defined as SBP within 5 mmHg of systolic Prest. We also looked at stable SBP_10mmHg_ (SBP within 10 mmHg). The variability of the automatic blood pressure monitor is expected to be 5 mmHg according to the manufacturer.

#### Prediction of the number of patients considered as hypertensive at various resting times

A patient is considered as having hypertension when measured SBP is equal or above 140 mmHg. To investigate the impact of resting time on the proportion of subjects predicted as hypertensive, we simulated 1000 replicates of the original datasets by sampling parameters from the final model estimates, and computed the corresponding proportion of subjects for various measurements (single measurement or mean of two points 1 or 2 mn apart)^12,15^. We also report the associated prediction intervals.

### Availability of data and materials

The datasets generated and analyzed during the current study are not publicly available but are available from the corresponding author on reasonable request.

## Electronic supplementary material

Supplementary Information
