# Supplementary material for: A minimal resting time of 25 min is needed before measuring stabilized blood pressure in subjects addressed for vascular investigations
Source: Sci Rep. 2017 Oct 10;7:12893. doi: 10.1038/s41598-017-12775-9 (PMC5635024; doi:10.1038/s41598-017-12775-9)
Supplement: Supplementary file 1 — Supplementary Information [file 41598_2017_12775_MOESM1_ESM.doc]

**SUPPLEMENTAL MATERIALS**

Title: A minimal resting time of 25 min is needed before measuring stabilized blood pressure in subjects addressed for vascular investigations

Authors list: Guillaume MAHE, Emmanuelle COMETS, Aziz NOUNI, François PAILLARD, Caroline DOURMAP, Alexis LE FAUCHEUR, Vincent JAQUINANDI.

**Methods**

***Data***

The main analysis was performed for systolic Blood Pressure (BP). The guidelines recommend measuring BP in the two arms and using the highest value. Therefore in the present analysis, we followed for each subject BP in the arm with the highest measurement at the first measurement in the study (t=1 min). A second analysis was performed for the diastolic BP measured on the same arm for each subject.

Data management, descriptive statistics, and basic statistical tests were performed using the statistical software R (R Core Team 2016). Unless otherwise specified, a p-value of 0.05 was considered significant for statistical tests.

***Model building***

The data was analysed using non-linear mixed effect regression. The statistical model for jth observation in ith subject y_ij_ in this model is written as follows:

y_ij_ = P(t_ij_, ϕ_i_) + ε_ij_ (1)

where ε represents the measurement error and is assumed to follow a normal distribution (ε_ij_~ N(0, g(t_ij_, ϕ_i_), σ)), with a variance that can depend on the model predictions and is modelled via a function g.

The function f in equation (1), termed structural model, represents the evolution of BP with time. The shape of the decrease in BP (Figure 1) suggested an exponential decrease, and we used the following function to describe it:

P(t) = Prest_i_ (1+dP_i_ exp(-k_i_ t_ij_)) (2)

f is a function of 3 parameters: Prest is the resting blood pressure representing the BP reached after a long rest, dP is the relative difference between the baseline pressure (before the subject sits or lays down), and k is a rate constant measuring the speed at which BP stabilises. In equation (1), ϕ_i_ is the individual (subject specific) vector of parameters (Prest_i_, dP_i_, k_i_). The interindividual variability (IIV) in non-linear mixed effect models is carried by the distribution of the parameters, which was assumed to be log-normal. For instance the variability model for Prest reads:

Prest_i_ = Prest e ^ηi^ (3)

where Prest is the population parameter, representing a fixed effect common to the entire population, and ηi is a subject-specific random effect. The distribution of the random effect was assumed to be jointly normal, and was characterised by a variance-covariance matrix Ω.

Parameter estimation was performed using the SAEM algorithm^1^, implemented in the Monolix software version 2016R1^2^. For model building, we first tested various functional forms for f (structural model) besides the exponential model in equation (1), then we selected an appropriate error model (function g). During this step, model selection was based on the log-likelihood ratio test (LRT) for nested models, and Bayesian Information Criterion (BIC) for non-nested models. With this base model, we then explored parameter-covariate relationships through a stepwise approach. Continuous covariates, for instance Age, were centered with respect to their median value in the population, and entered in equation (3) as:

Prest_i_ = Prest (Age/median(Age))^βPrest, Age^ e ^ηi^ (4)

where β_Prest,Age_ represents the effect of Age on the parameter Prest. Binary covariates were also entered multiplicatively, with equation (3) in the reference category (eg Male) becoming for the alternative category (eg Female):

Prest_i_ = Prest e ^βPrest, Gender^ e ^ηi^ (5)

In the first step, we included all potential relationships in a full model, and we used the Wald test to determine which covariates to keep: covariates with a p-value larger than 0.2 were removed and the model was re-run. If there were covariates with p-values higher than 0.2, we then removed the covariate with the highest p-value (most non-significant), and repeated the process iteratively until all covariates had p-values lower than 0.2. For this first step, we needed to divide the candidate covariates in two sets (demographic+comedications and clinical conditions, with Gender and Age in both sets to avoid confounding) because of the size of the dataset and the correlations between the covariates, and only covariates present in at least 10 subjects were considered. Later, only significant covariates were loaded in the Monolix dataset which alleviated this technical issue. In the second step, we combined the two submodels where all p-values were lower than 0.2 into one, and repeated the stepwise descending selection with a more stringent criterion until all covariates remaining in the model were significant with a p-value for the Wald test equal to 0.05.

The entire covariate selection was performed assuming a full variance-covariance matrix to allow maximum flexibility in the search for covariate relationships. The covariance structure was tested again in the final model.

***Model evaluation***

Model evaluation was performed through diagnostic graphs, including plots of the observed versus predicted BP, visual predictive checks (VPC)^3^ and normalised prediction distribution errors (npde)^4^. We also evaluated the robustness of the model estimates for the final model through the run assessment feature in Monolix, by performing 10 runs with different seeds and different starting estimates. Covariate effects which were not significant in at least 8 out of 10 runs were removed from the model and the parameters re-estimated, so that the final model retained only robust covariate effects.

***Investigating model type***

Individual profiles showed a diversity of evolutions with time, with some subjects showing only seemingly random fluctuations and others exhibiting a linear decrease without a visible plateau. We investigated this through mixtures of models in the initial analyses, but these models are sensitive to initial conditions and we could not obtain a good fit. To further investigate this issue, we created a covariate reflecting the shape of the decrease, through individual non-linear regression (NLR). NLR was performed for each subject to assess whether a non-linear or linear model fit best the data collected, and whether the pressure could be considered as stable (P(t)=Prest, constant over the measurement period). Model selection was performed individually using BIC. For each subject, we defined a covariate modelType, with three possible values (constant, linear or non-linear), depending on the model selected through RNL.

The covariate modelType was then used to refine the model. We tested different models using the same criteria as in the main model building in the previous section, including models with different shapes mixing constant BP or delayed decrease, and models with modelType as a covariate, where the typical value of the parameter and possibly its variability depend on modelType. A model with modelType as a covariate on the 3 parameters but not their variability was found to perform best in terms of statistical criteria. We then investigated the other covariates using the same approach as in the model building strategy described above.

***Time to reach a stable blood pressure***

The time to reach a given blood pressure can be derived from the asymptotic model given in equation (1). For instance, the time to reach a BP within 5mmHg of the resting blood pressure can be computed as:

T_Prest+5_ = (log(Prest)+log(dP)-log(5))/k

The distribution of given predicted resting times was obtained by simulations. To account for the covariate distribution, we simulated 1000 sets of parameters (Prest, dP, k) using the population estimates and the covariates in the observed population, yielding 1000*N virtual subjects. We removed outliers, identified as simulated subjects for which one of the simulated parameters was outside the range of individual estimates from the original analysis, and obtained the distribution of T_Prest+5_ (time to reach a BP within 5mmHg of Prest) and T_Prest+10_ (time to reach a BP within 10mmHg of Prest). These distributions were used to assess the time at which 90% of the population can be expected to have reached a stable blood pressure (respectively T_Prest+5,90%_ and T_Prest+10, 90%_). The simulations were performed with the final model in each analysis.

***Proportion of the population considered as hypertensive***

We evaluated the guidelines recommendations in our population through simulations. The guidelines recommend to take two measurements 1 or 2min apart after 3 or 5min rest, averaging the two measurements^5,6^. We computed the proportion of subjects considered as hypertensive (pressure higher than 140mmHg) according to the recommendations, and compared them to the average of two later time points (1 and 2min from T_Prest+5,90%_ and T_Prest+10, 90%_). As in the previous section, we simulated 1000 datasets with the same covariate structure as the original dataset (199 subjects), including residual error to mimic the observation process.

We evaluated the mean and prediction interval of the proportion of hypertensive subjects according to these different metrics and we compared them to the same proportion predicted using either a single time-point (between 3 and 27 min) or the mean of 2 measurements taken 1 or 2 min apart but at later time-points (10, 15, 20 and 25 min).

**Results**

***Study population***

Table 1 shows the characteristics of the population studied in this analysis, which included 101 subjects in the reclining position (51%) and 98 subjects in the sitting position (49%). BP was followed on the arm for which the highest value was observed at the time of the first measurement, which was the left arm in n=106 subjects (53%) and the right arm in n=93 subjects (47%).

***Model building***

We tested different structural models to fit the data in this work, including biexponential and delayed exponential decrease. We also tested mixture distributions on the different parameters in the model, but we could not identify the parameters in the mixture. Finally, we tested mixture of models, which combine constant and exponential, or several exponential models. In the end, an exponential decrease was found to fit the data best according to the Bayesian Information Criterion. We tested additive, proportional and combined error model, and found the best model to be a proportional error model, where the standard deviation of the error term is proportional to the model prediction. The next step was to build the covariate model. Covariate exploration showed a weak relationship between parameters and several covariates, but after stepwise selection, only the following 5 covariates remained in the final model: Age, cardiac insufficiency, heart rate, recent coffee intake and betablocker agents. The run assessment feature (10 runs performed with different seeds and different initial conditions) showed that the parameter estimates were robust across the different runs (Figure S1). Finally, because of the high estimation error for the correlation between the random effects for Prest and dP, and because the covariates explain part of the variability, the variability structure was tested again and the final model was run with a diagonal variance-covariance matrix.

Parameter estimates for this final model are reported in Table 1 (main text), with a resting BP estimated at 131mmHg in this population. Baseline BP was estimated to be 25% higher on average than resting BP. k, the rate constant governing the time to stabilisation was estimated at 0.4 min^-1^, with a large variability in the population (84%). k is related to the half-life by the equation t_12_=log(2)/k; t_12_ represents the time necessary for the BP to decrease by half the difference between this BP and Prest, and we estimated to be 1.7min in the typical subject, with 95% of the population expected to have values between 0.33 and 8.9min (interindividual variability). Age was found to be positively correlated both to rest BP and to the change from baseline, while cardiac insufficiency was associated with lower Prest as well as a slower stabilisation. Recent coffee intake increased dP, the difference between baseline and rest BP, without affecting Prest, suggesting it increases the baseline BP. On a side-note, hypertension did not remain in the final model as a covariate explaining any of the model parameters, and especially Prest, which could be attributed to the confounding effect of treatment as most patients with hypertension received medication.

Figure 2 (main text) shows individual model fits for 12 randomly selected patients, demonstrating a good model adequacy. Figure S2 shows diagnostic plots and Figure S3 presents the VPC.

**Figure S1** – *Summary of the run assessment step, showing the estimates of each parameter and their estimation error (whisker plot) across the 10 runs.*

**
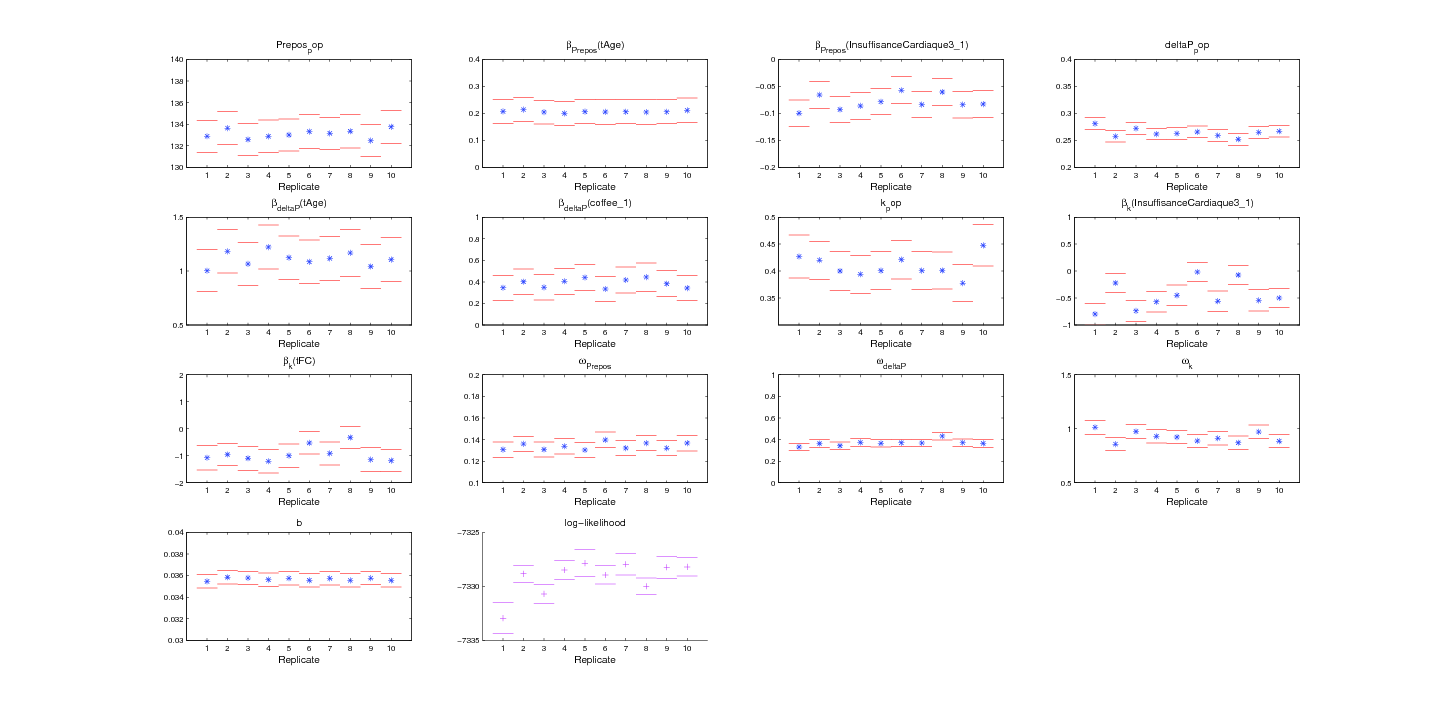
**


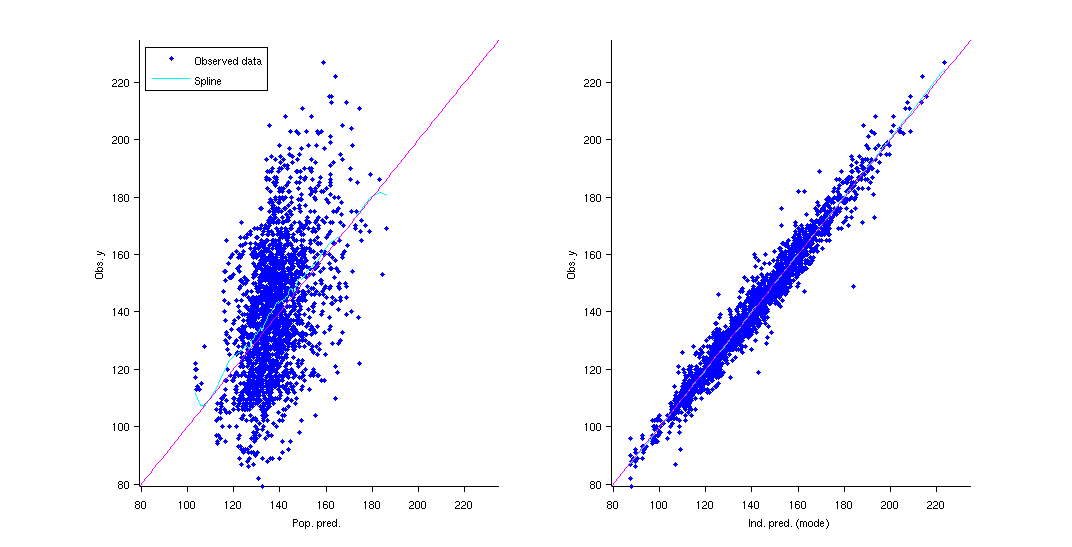
**Figure S2** – *Diagnostic graphs for the final model for SBP. Top: Observed versus predicted (left: population predictions, right: individual predictions). Bottom: normalised prediction distribution errors versus time (left) and predictions (right).*


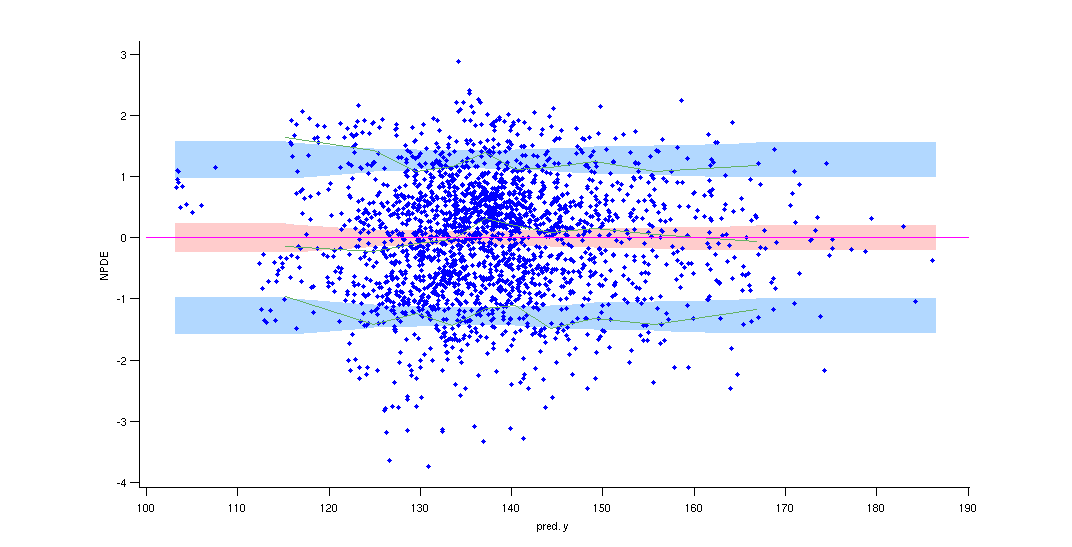

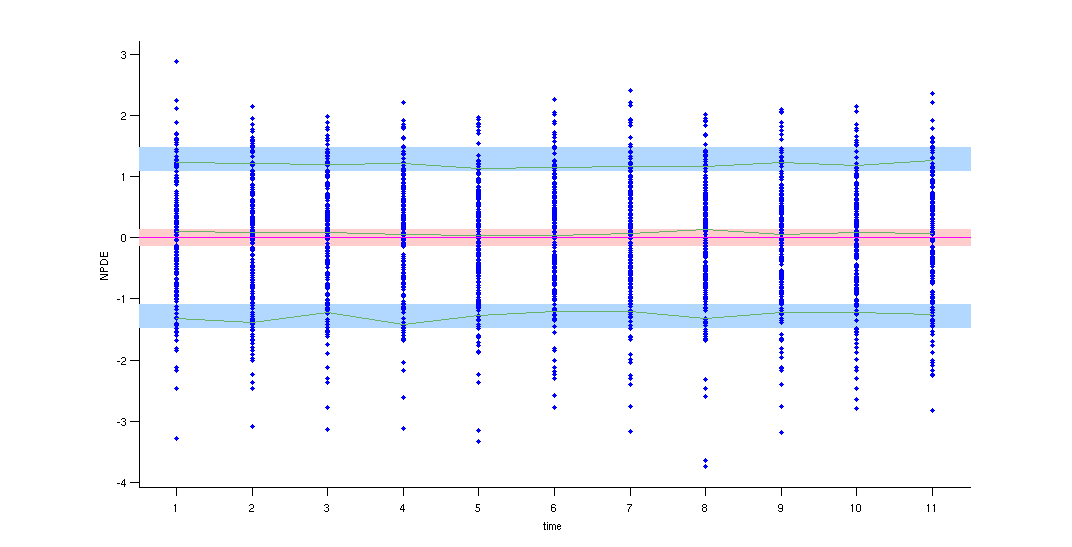


**Figure S3 –** *Visual Predictive Check (VPC) for the final model for SBP, obtained using 1000 simulations under the model. Green lines represent the empirical percentiles computed for the observed data.*

***
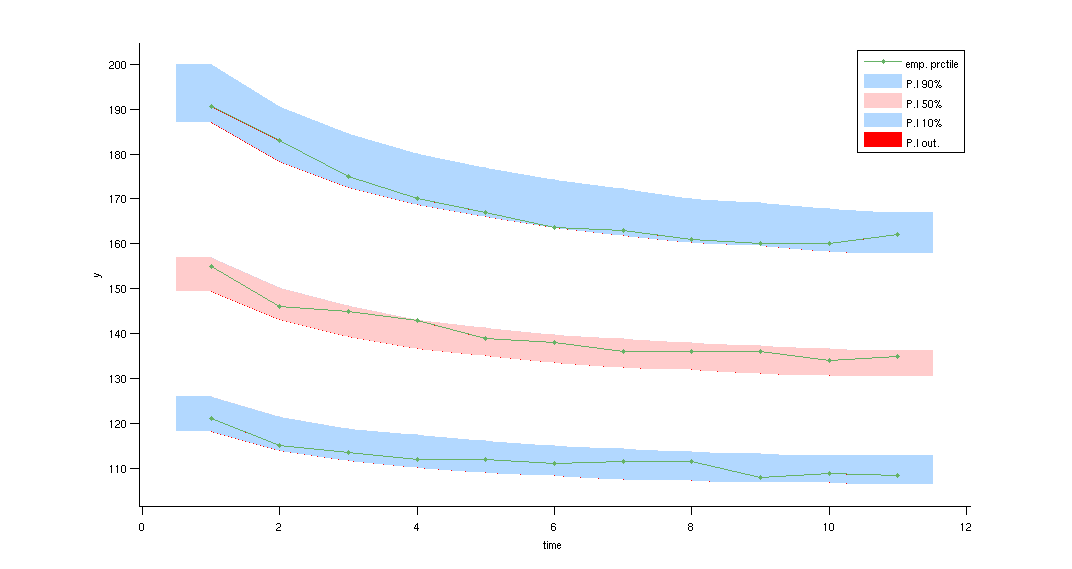
***

***Model building taking into account model type***

An additional analysis was undertaken to reduce the variability in k by defining a covariate reflecting the shape of the decrease. Individual non-linear regression showed that a non-linear model fit the data best in 128 subjects (64%), while 44 (22%) had a linear decrease and 24 (14%) did not exhibit significant variation. We defined a covariate modelType with 3 categories (respectively regular, slow and fast), reflecting the rate of decrease of BP observed in these 3 groups. There was no clear relationship between modelType and other covariates in the dataset when correlations were tested using a Fisher test, with a Bonferroni correction to account for multiple tests. The covariate modelType was included in the model, and was found to impact both the Prest and the time to stabilisation. The other covariates influencing Prest and dP in the first analysis were again significant when modelType was accounted for, and the parameter estimates for these covariate effects was similar in both models.

There was no significant impact of modelType on dP, and we also tested whether the parameters influenced by modelType had different variances in the 3 groups, but this was not significant. The parameter estimates for the final model with modelType included as a covariate are shown in table S1.

Including modelType in the model led to the identification of 3 groups of subjects with different values of k: fast, regular and slow stabilisers. The majority of subjects had a systolic Prest around 136mmHg, with an estimate of k similar to the previous analysis (k=0.49 min^-1^, yielding a half-life of 1.4 min). Fast stabilisers have a typical value of k 5 times higher than the majority of the population (k=2.56 min^-1^, half-life of 27s), and their BP essentially fluctuates around its value at rest during the entire study. On the other hand, slow stabilisers take about 5 times longer than the regular population to stabilise, showing a much slower decrease (k=0.08 min^-1^) with a half-life of 9 min. They also were found to have lower Prest than the other two groups (115mmHg), and constraining to the same Prest in the three groups performed much worse, although this could be a sample effect or influenced by identifiability issues and would require confirmation in a population followed for a longer duration than in the present study. Taking into account individual differences in the shape of the decline allowed to reduce the variability in k by 40% compared to the model without modelType. The other covariates in the model were similar compared to model without modelType for Prest and dP, except for an additional gender effect found of dP. The two covariates dyspnea and cardiac rate impacting k in the previous analysis were not retained in the final model, possibly as a result of explaining the variability in this parameter through modelType.

Figure S4 shows the VPC obtained for this model, stratified across modelType. For all groups, the diagnostic graphs showed a good model adequacy.

**Table S1:** *Parameter estimates for final model(SBP) with model type included as a covariate. RSE stands for relative estimation error, IIV denotes the interindividual variability, quantified by the standard deviation (SD) of the random effect associated to the population parameter. Dyspnea stands for subjects who declared to have dyspnea when walking.*

| **Parameter** | **Estimate (RSE %)** | **SD % (RSE %)** |
| --- | --- | --- |
| **Prest (mmHg)** | 136.60 (1) | 13 (5) |
| **β_Prest, Age_ (-)** | 0.20 (25) | - |
| **β_Prest, Dyspnea_ (-)** | -0.055 (44) | - |
| **β_Prest, slow_ (-)** | -0.17 (16) | - |
| **β_Prest, fast_ (-)** | -0.02 (128) | - |
| **dP (-)** | 0.33 (5) | 28 (11) |
| **β_dP, Female_ (-)** | -0.16 (41) | - |
| **β_dP, Age_ (-)** | 0.77 (23) | - |
| **β_dP, Recent \; coffee_ (-)** | 0.37 (27) | - |
| **k (min^-1^)** | 0.49 (6) | 55 (8) |
| **β_k, slow_ (-)** | -1.86 (8) | - |
| **β_k, fast_ (-)** | 1.66 (11) | - |
| **a (mmHg)** | 1.13 (45) | - |
| **σ (-)** | 0.027 (14) | - |

**Figure S4** – *Visual Predictive Check (VPC) for the final model stratified by modelType (left: regular, middle: slow, right: fast stabilisers).*

***
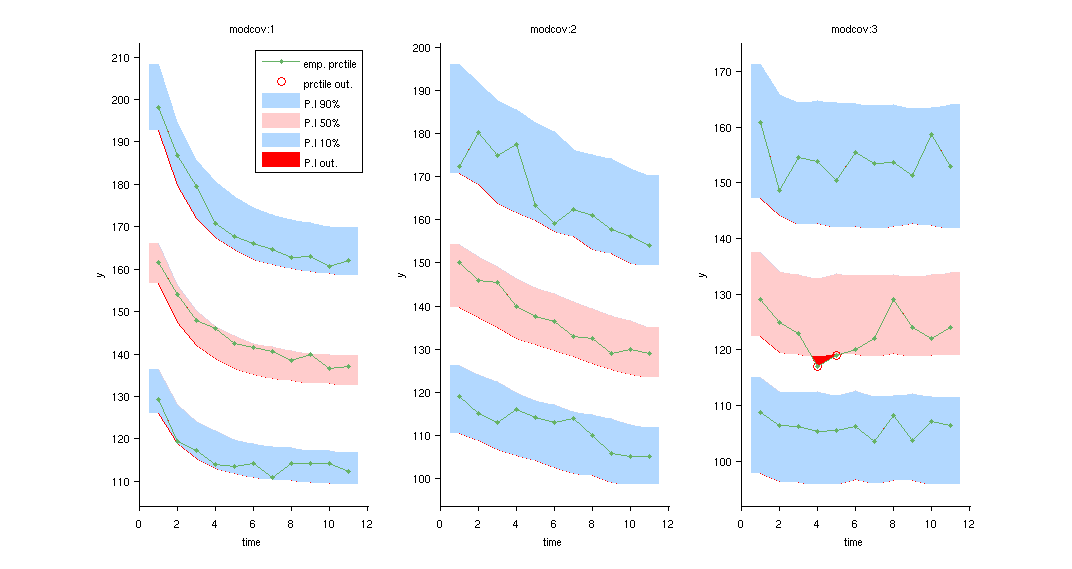
***

***Modelling diastolic blood pressure***

We modelled diastolic blood pressure in a similar way as systolic blood pressure, in a separate analysis. As for systolic BP, we first modelled the decrease of diastolic BP assuming a common log-normal distribution for all subjects, and in a second step, we defined as previously a subject-specific model type using individual non-linear regression.

The final model estimates obtained with these two analyses are reported in Table S2. The diastolic model type was different from the systolic model type in 134 subjects (67%), with 78 subjects showing a flat profile (39%) compared to 27 for systolic BP, and 80 (40%) stabilising slowly compared to 44 for systolic BP. However, for diastolic BP, there was no significant difference in the final model between the slow and the regular stabilisers, so we regrouped them in one group versus the subjects who did not show significant fluctuations. The analyses with or without model type yielded similar estimates of Prest and dP, but in the second analysis the estimate of k in Table S2 represents the rate of decrease of systolic BP in the group of slow or normal stabilisers and is therefore lower. Dyspnea was the only covariate to remain in both models on Prest, while most other covariates disappeared with the introduction of model type in the model. Also, contrary to the analysis in systolic BP, the introduction of model type did not appear to explain interindividual variabiltiy much, as the IIV especially on k remained extremely high. The estimate of diastolic Prest was around 70mmHg, about 40mmHg lower than the estimate of systolic BP. The estimate of dP was around 0.2, so that we expect on average a difference of 14mmHg (0.2*70) between Prest and the baseline systolic BP.

Figure S5 shows the VPC obtained for respectively the model without (top) and with model type (bottom), for the entire population.

**Table S2:** *Parameter estimates for the models describing the time course of diastolic BP, for the models without (left) and with (right) model type included as a covariate. RSE stands for relative estimation error, IIV denotes the interindividual variability, quantified by the standard deviation (SD) of the random effect associated to the population parameter. Dyspnea stands for subjects who declared to have dyspnea when walking.*

|  | **Model without model type** | | **Model with model type** | |
| --- | --- | --- | --- | --- |
| **Parameter** | **Estimate (RSE %)** | **SD % (RSE %)** | **Estimate (RSE %)** | **SD % (RSE %)** |
| **Prest (mmHg)** | 71.1 (1) | 14 (5) | 68.5 (1) | 12 (6) |
| **β_Prest, Age_ (-)** | -0.10 (46) | - | - | - |
| **β_Prest, Dyspnea_ (-)** | -0.04 (60) | - | -0.07 (31) | - |
| **β_Prest, fast stabilisers_ (-)** | - | - | 0.06 (28) | - |
| **dP (-)** | 0.17 (10) | 71 (12) | 0.23 (7) | 47 (14) |
| **β_dP, Dyspnea_ (-)** | 1.12 (23) | - | - | - |
| **β_dP, Atrial fibrillation_ (-)** | - | - | 0.75 (35) | - |
| **k (min^-1^)** | 0.71 (17) | 160 (7) | 0.27 (19) | 187 (7) |
| **β_k, Age_ (-)** | -3.71 (21) | - | - | - |
| **β_k, Dyspnea_ (-)** | 1.54 (23) | - | - | - |
| **β_k, Infarctus_ (-)** | 0.72 (55) | - | - | - |
| **β_k, fast stabilisers_ (-)** | - | - | 4.04 | (18) |
| **σ (-)** | 0.045 (2) | - | 0.045 (2) | - |


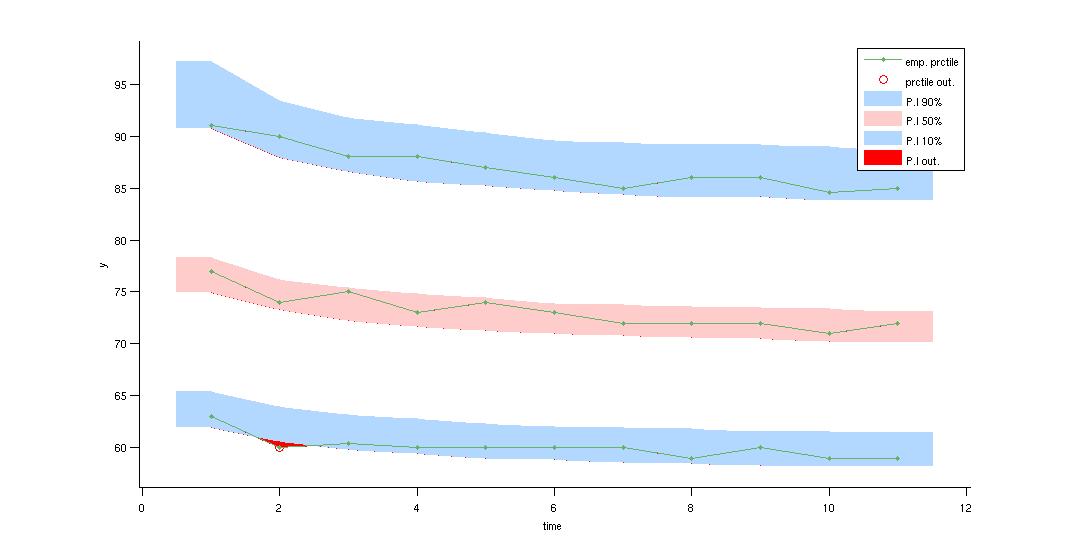
***Figure S5 –*** *Visual Predictive Check (VPC) for the final models for the analysis of diastolic BP, without (top) and with (bottom) the model type covariate defined by individual non linear regression analysis. PI stands for prediction interval. The pink band denotes the PI around the median, and the blue bands the PIs around the 10 and 90% quantiles of the predicted BP. Empirical percentiles of the observed DBP are shown as green lines and should remain within their corresponding PI under the model. Red bands indicate regions where the observed percentiles deviate from model predictions.*

*
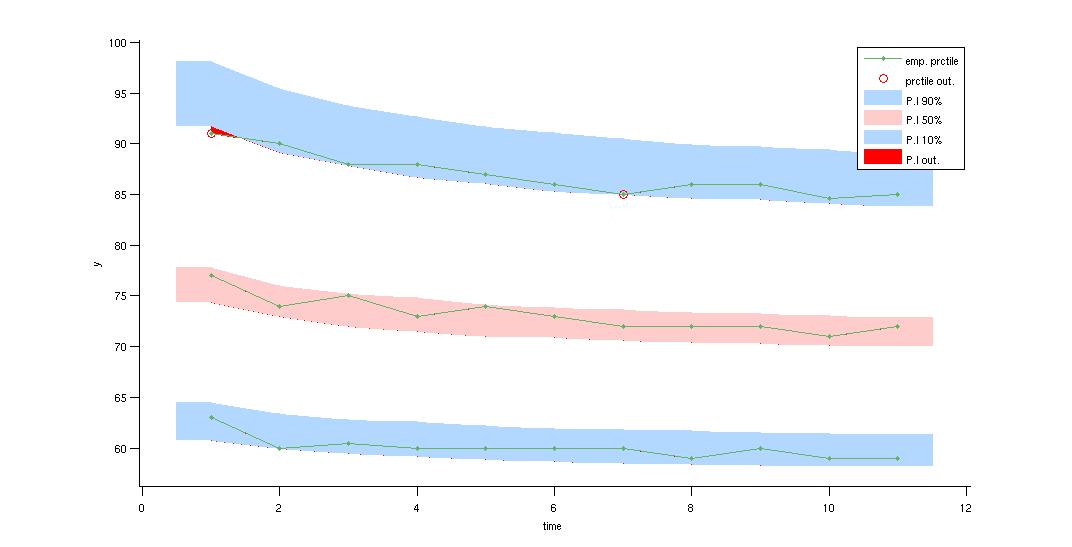
*

***Time to reach a stable systolic blood pressure***

Table S3 shows the predicted time at which a given percentage (50 to 95%) of the population is predicted to reach a systolic BP within 5mmHg of Prest, for the models with and without modelType included. For instance, using the model without modelType, we predict that 75% of the population will reach a stable BP in about 11 min, while only 50% of the population can be expected to be within 5mmHg of Prest after 5.5 min, and it may take up to 24 min to ensure 90 % of subjects to be stabilised. On the other hand, 90% of the population reaches a BP within 10mmHg of Prest in 15 min.

With the model including modelType, we predict similar times to stabilisation up to 90% of the population. For this model, the next lines in the table show the proportions stratified by group. 90% of the subjects in the fast group, who are nearly stable throughout the study, are therefore predicted to be within Prest+5mmHg after 1.5min, and subjects in the regular group are also almost all at rest after 9min. However, subjects in the slow group take much longer to stabilise, and since they form around 22% of our sample, the time to achieve a stable resting BP increases overall. The values predicted for this group however should be taken with some caution as we lack late measurements to extrapolate so far outside the measurement period, but the trend in the data was clearly not showing stabilisation in the 11 minutes of the observation period for this group (Figure S4).

The figures shown in table S3 reflect the predictions of the model taking into account the distribution of covariates in our study, through the estimates of covariate effects, so that the times to stabilisation reflect in part the structure of the population in our study. However, our purpose in obtaining these estimates was to determine the times at which to evaluate the proportion of hypertensive subjects in the next section.

**Table S3:** *Predictions of time (in mn) at which a given percentage of the population reaches a value of SBP within Prest+5mmHg and within Prest+10mmHg in the final model in both analyses, stratified by group in the model with modelType as a covariate. The numbers were obtained based on 1000 replicates of the original dataset, and account for the distribution of covariates in this study.*

| **Percentage of the population** | **50%** | **75%** | **80%** | **85%** | **90%** | **95%** |
| --- | --- | --- | --- | --- | --- | --- |
| **Time to reach SBP within Prest+5mmHg** | | |  |  |  |  |
| **Model without modelType** | 5.56 | 11.22 | 13.66 | 17.32 | 23.83 | 40.23 |
| **Model with modelType** | 4.75 | 9.59 | 12.59 | 17.52 | 24.10 | 33.58 |
| **Regular group** | 4.35 | 6.42 | 7.06 | 7.88 | 9.09 | 11.16 |
| **Slow group** | 25.21 | 37.55 | 41.19 | 45.86 | 52.78 | 64.78 |
| **Fast group** | 0.71 | 1.06 | 1.17 | 1.31 | 1.52 | 1.88 |
| **Time to reach SBP within Prest+10mmHg** | | |  |  |  |  |
| **Model without modelType** | 3.39 | 7.04 | 8.59 | 10.98 | 15.12 | 25.83 |
| **Model with modelType** | 3.18 | 6.42 | 8.16 | 11.05 | 15.36 | 21.94 |
| **Regular group** | 2.91 | 4.38 | 4.84 | 5.43 | 6.32 | 7.82 |
| **Slow group** | 15.93 | 24.37 | 26.99 | 30.35 | 35.27 | 43.48 |
| **Fast group** | 0.44 | 0.68 | 0.76 | 0.85 | 1.00 | 1.26 |

***Proportion of the population predicted as hypertensive***

Table S3 suggests that measuring SBP according to guidelines may be too early for at least half of the population. To evaluate the impact of measurement time on the estimation of the proportion of subjects diagnosed as hypertensive (SBP higher than 140), we simulated the time course of SBP using the design (number of subjects and covariate distribution) of our original dataset over a period of 25 min to cover the predicted times in table S3 (T_Prest+5,90%_, rounded to 25 min, and T_Prest+10, 90%_, rounded to 15 min). We then computed the proportion of subjects diagnosed as hypertensive at different time points, either single time points or the mean of two time points 1 or 2 min apart. This simulation was repeated 1000 times to obtain a prediction interval taking into account interindividual and residual variability.

The result is shown in Figure 3 (main text) for the model without modelType, but a very similar plot (not shown) was obtained for the model including that covariate, suggesting our results are robust to the choice of the model. Note that the scales for the X-axes in the two figures (left: when considering only one measurement and right: when considering the mean of two measurements) are not comparable, as the X-axis in the left figure represents time, while in the right figure it represents the mean at different time points. Figure 3 shows that measurements of SBP taken after only 3 or 5 min rest, single or averaged, tend to overpredict the proportion of hypertensive, which stabilizes only after 15 to 20 min. We can also note from the simulation results that the time interval (1 or 2 min) has more impact on early compared to late measurements.

The width of the prediction intervals reflects the small sample size (n=199), and would warrant further exploration in an appropriately powered study. However, our results suggest that even in a rather small study of 199 subjects, the proportion of hypertensive subjects could decrease from 53% [46-60%] to 40.5% [34-47%] and 38% [33-44%] whether subjects are allowed to rest 3, 15 or 25 min before BP is measured. In our sample, there was indeed a significant difference between the proportion of subjects with SBP over 140 mmHg when taking the mean of the measurements at times 3 and 5 compared to the mean at times 9 and 11 (55% versus 42%, p=5.10-6 according to a McNemar test for correlated proportions). The observed proportion dropped to 44% when considering the mean at times 5 and 7 (NS compared to the mean of measurements at 9 and 11 min). For completion, table S3 shows the proportion of hypertensive subjects predicted using the mean of two observations taken 2 min apart, with the first of the two measurements taken at 3, 5, 10, 15, 20 and 25 min; the results are shown as mean and prediction interval obtained over the 1000 simulations.

**Table S3**: *Predicted proportion of hypertensive subjects according to the mean of two observations, using 1000 datasets simulated under the final model without modelType with the same covariate structure as the original dataset.*

| **Measurement times (min)** | 3+2 | 5+2 | 10+2 | 15+2 | 20+2 | 25+2 |
| --- | --- | --- | --- | --- | --- | --- |
| **Predicted proportion of hypertensive subjects** | 50 [44-57] | 44 [38-50] | 38 [31-44] | 35 [29-41] | 34 [28-40] | 33 [27-39] |

**References for Supplemental material**

1. Kuhn, E. & Lavielle, M. Maximum likelihood estimation in nonlinear mixed effects models. *Comput. Stat. Data Anal.* **49,** 1020–1038 (2005).

2. Lavielle, M. *Mixed effects models for the population approach: models, tasks, methods and tools*. (Taylor & Francis, 2014).

3. Holford, N. The visual predictive check - superiority to standard diagnostic (Rorschach) plots. (2005).

4. Brendel, K., Comets, E., Laffont, C., Laveille, C. & Mentré, F. Metrics for external model evaluation with an application to the population pharmacokinetics of gliclazide. *Pharm. Res.* **23,** 2036–2049 (2006).

5. Mancia, G. *et al.* 2013 ESH/ESC guidelines for the management of arterial hypertension: the Task Force for the Management of Arterial Hypertension of the European Society of Hypertension (ESH) and of the European Society of Cardiology (ESC). *Eur. Heart J.* **34,** 2159–2219 (2013).

6. Pickering, T. G. *et al.* Recommendations for blood pressure measurement in humans and experimental animals: part 1: blood pressure measurement in humans: a statement for professionals from the Subcommittee of Professional and Public Education of the American Heart Association Council on High Blood Pressure Research. *Circulation* **111,** 697–716 (2005).
